# Supplementary material for: Genomewide phenotypic analysis of growth, cell morphogenesis, and cell cycle events in Escherichia coli
Source: Mol Syst Biol. 2018 Jun 25;14(6):e7573. doi: 10.15252/msb.20177573 (PMC6018989; doi:10.15252/msb.20177573)
Supplement: Supplementary file 1 — Appendix [file MSB-14-e7573-s001.docx]

APPENDIX

Genome-wide phenotypic analysis of growth, cell morphogenesis and cell cycle events in *Escherichia coli*

Manuel Campos, Sander K. Govers, Irnov Irnov, Genevieve S. Dobihal, François Cornet and Christine Jacobs-Wagner

Table of Contents

[Appendix Figures S1-S8 2](#_Toc510602909)

[Appendix Tables 12](#_Toc510602910)

[Appendix Parameters 17](#_Toc510602911)

[References 20](#_Toc510602912)

# Appendix Figures S1-S8


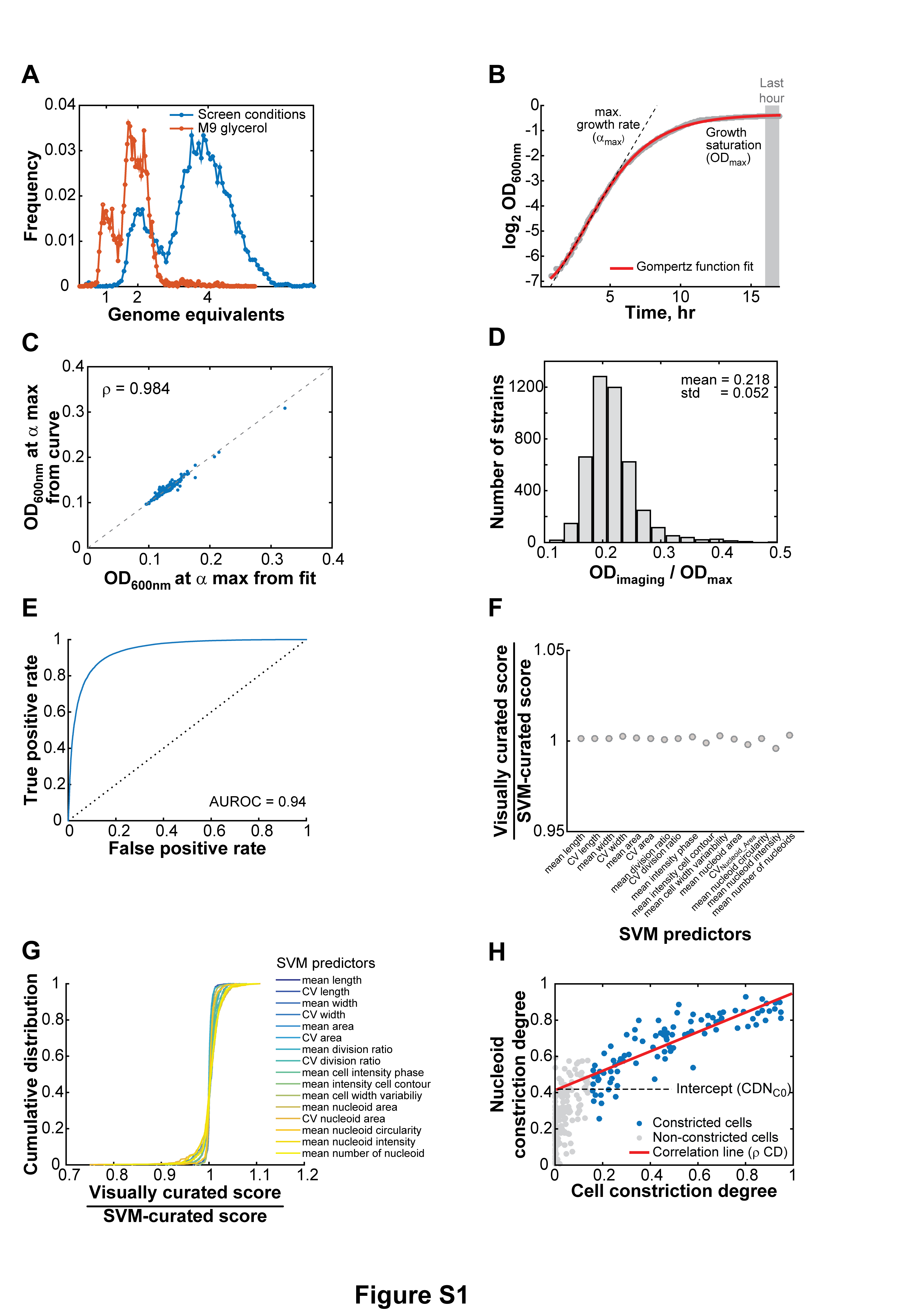
**Appendix Figure S1. Feature determination and SVM model validation**.

**A.** Distribution of genome equivalents per cell determined by the signal intensity of DAPI DNA stain after a replication run-out experiment. BW25113 cells were grown at 30˚C either in M9 glycerol (poor nutrient condition – orange curve) or under the same growth conditions as for the screen (M9 glucose supplemented with casamino acids, a richer nutrient condition – blue curve), and then treated with rifampicin and cephalexin for 3 h to block cell division and prevent new rounds of DNA replication. Cells grown in M9 glycerol undergo a single cycle of DNA replication per division cycle (Cooper & Helmstetter, 1968; Wang et al, 2011). After rifampicin and cephalexin treatment, these cells contained either one or two chromosomes, and displayed a fluorescence level with DAPI corresponding to 1 and 2 genome equivalents. This calibration was used to determine the number of genomes per cell for cells growing under the richer nutrient conditions used for the screen.

**B.** Typical growth curve represented as the log_2_ of OD_600nm_ as a function of time. The red line shows the best Gompertz fit to the curve ($OD\left( t \right)=ae^{e^{\frac{{be}^{1}\left( c-t \right)}{a}+1}+d}$ where *t* is time and *a*, *b*, *c* and *d* are the four fitted parameters). The dotted line highlights the segment of maximal growth. The OD_600nm_ was averaged over the last hour of growth (gray box) to estimate the saturation level of the culture (OD_max_).

**C.** Scatter plot showing the relationship between the OD_600nm_ at α_max_ calculated from the growth curve and fitted curve. The correlation between both optical densities is high (ρ = 0.984, 95% CI [0.983, 0.985).

**D.** Histogram showing the ratio between the optical density at the time of sampling (OD_imaging_) and the optical density at saturation of the culture (OD_max_) measured from the growth curve. The mean value of this ratio is low (0.218 ± 0.052), indicating that Keio strains were imaged early in their population growth cycle.

**E.** AUROC curve (performance curve) related to the SVM model on the dataset that was not used to train the model. The dotted line y=x represents the expectation from a random classification.

**F.** Ratios between the mean values of each predictor for the visually-curated and SVM-curated datasets, showing the lack of bias.

**G.** Cumulative distributions of mean score ratios between visually- and SVM-curated datasets of mean and CV predictor values for the 419 strains with the most extreme phenotypes. The steepness of the curves shows that the SVM model performed well, even for strains with strong phenotypic defects.

**H.** Plot showing how two cell cycle features, the correlation between the degrees of constriction of the nucleoid and of the cell (ρ CD) and the projected degree of nucleoid constriction at the onset of cell constriction (CDN_C0_), were calculated using a WT culture as an example. The degree of constriction for both the nucleoid and the cell (considering only cells with a degree of cell constriction over 15%) were used to calculate their Pearson correlation coefficient (ρ CD). The correlation coefficient can be interpreted as the slope of the line passing through the data, and the intercept of this line with the y-axis provides the average degree of constriction of the nucleoid at the onset of cell constriction (CDN_C0_).


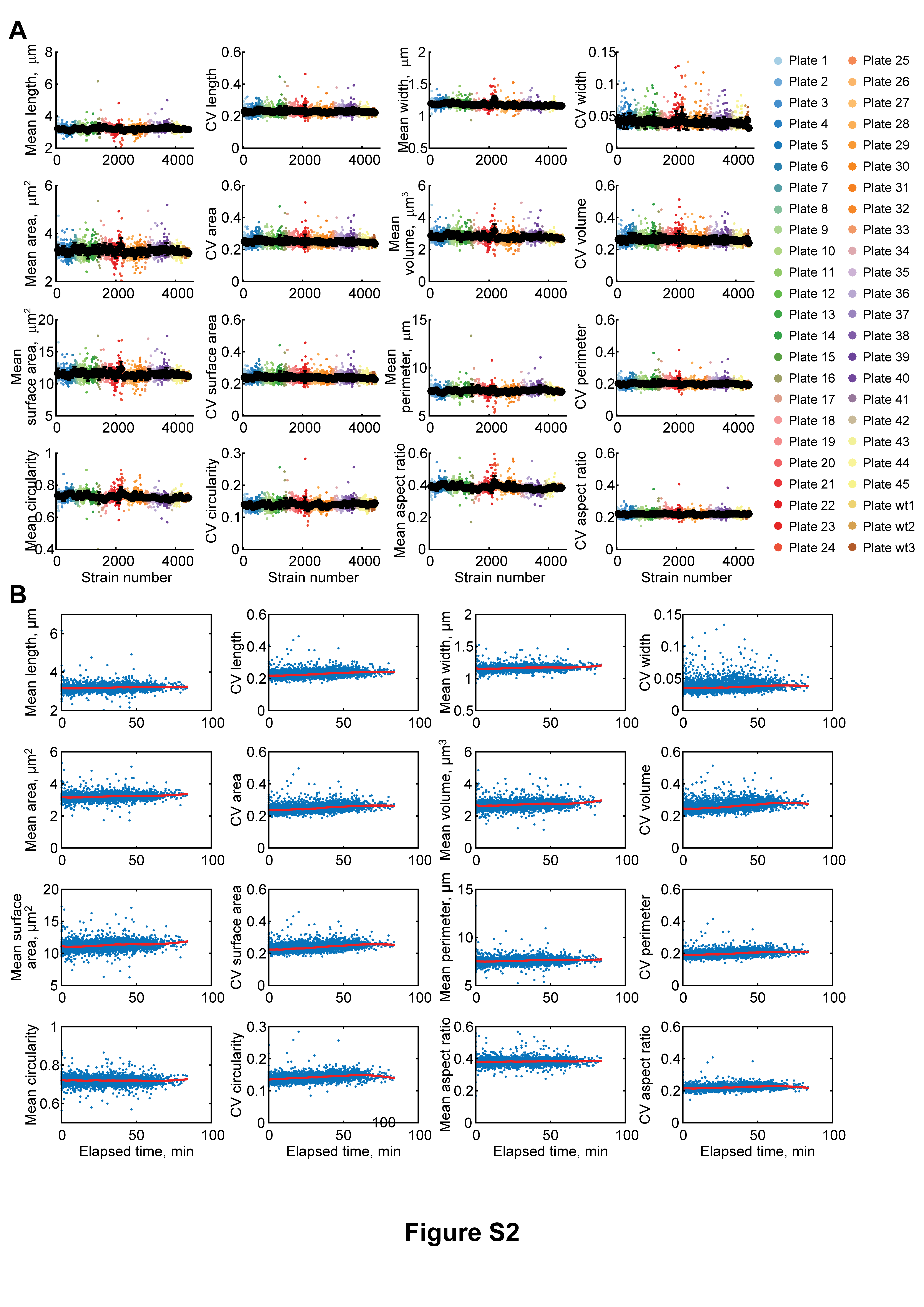


**Appendix Figure S2. Evaluation of positional and temporal biases related to imaging**.

**A.** Plate-by-plate normalization. Each plate is color-coded according to the 96-well plate number. The black dots represent the mean feature value per plate, ± standard deviation.


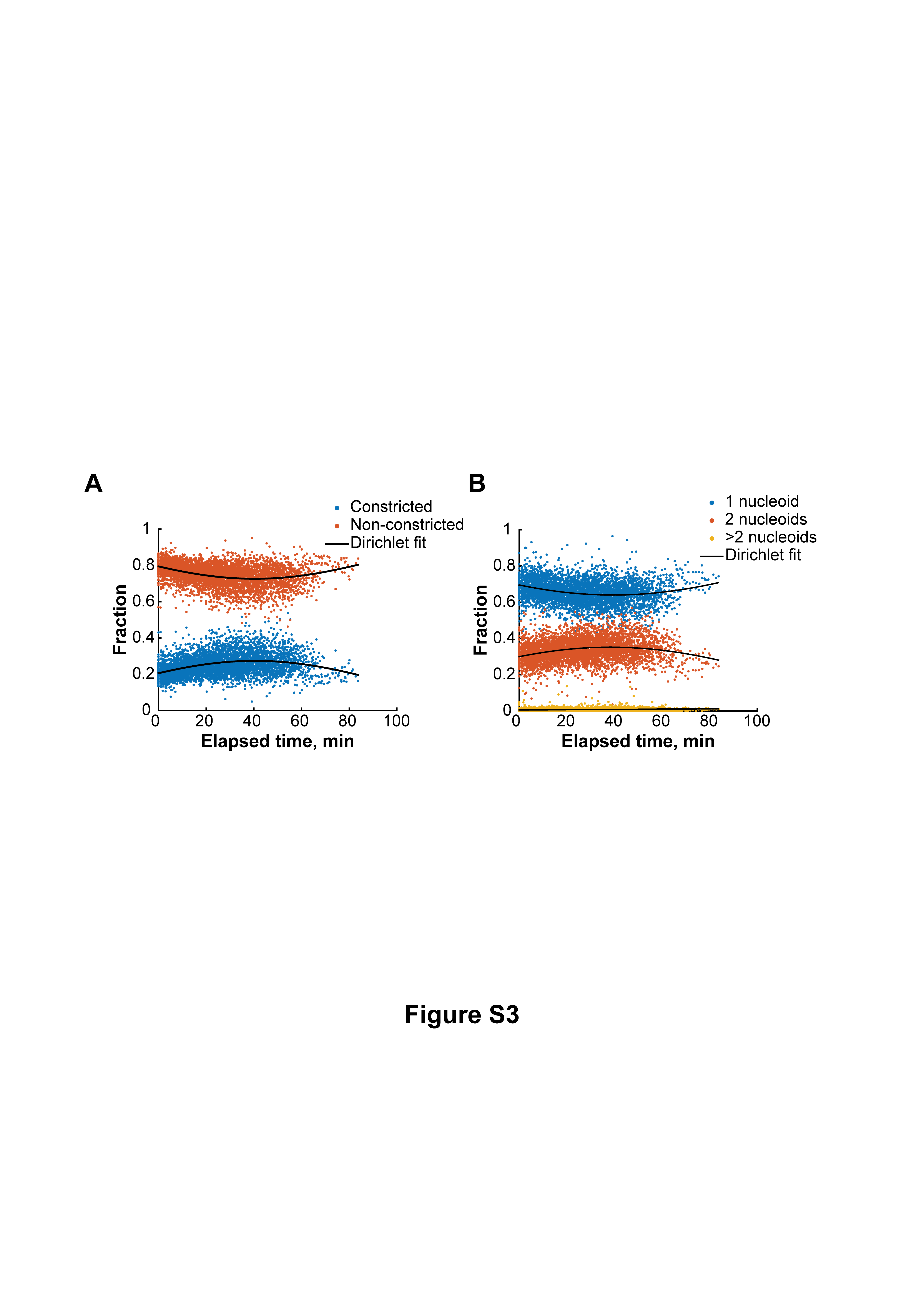
**B.** Scatter plots for each feature of all strains as a function of the time elapsed since spotting cells on the pad for imaging. The red line in each graph represents the smoothing spline (calculated with a span of 20%) that was used to correct any temporal bias.

**Appendix Figure S3. Temporal bias correction for proportional features**.

**A.** Scatter plot showing the proportions of constricting (blue) and non-constricting cells (red) for all strains (n = 4,227) as a function of the time elapsed between the time the cells were spotted on the pad and the time they were imaged. The solid black lines represent the correction factors over time, derived from a quadratic form Dirichlet regression to the data. The Dirichlet regression allows for the maintenance of the additivity of the proportion (Maier, 2014).

**B.** Same as in **A** for the complementary proportions of cells with 1 (blue), 2 (red) or more than 2 (yellow) nucleoids. The fitted model was quadratic for the first two features (1 and 2 nucleoids) and linear for the cells with more than 2 nucleoids.


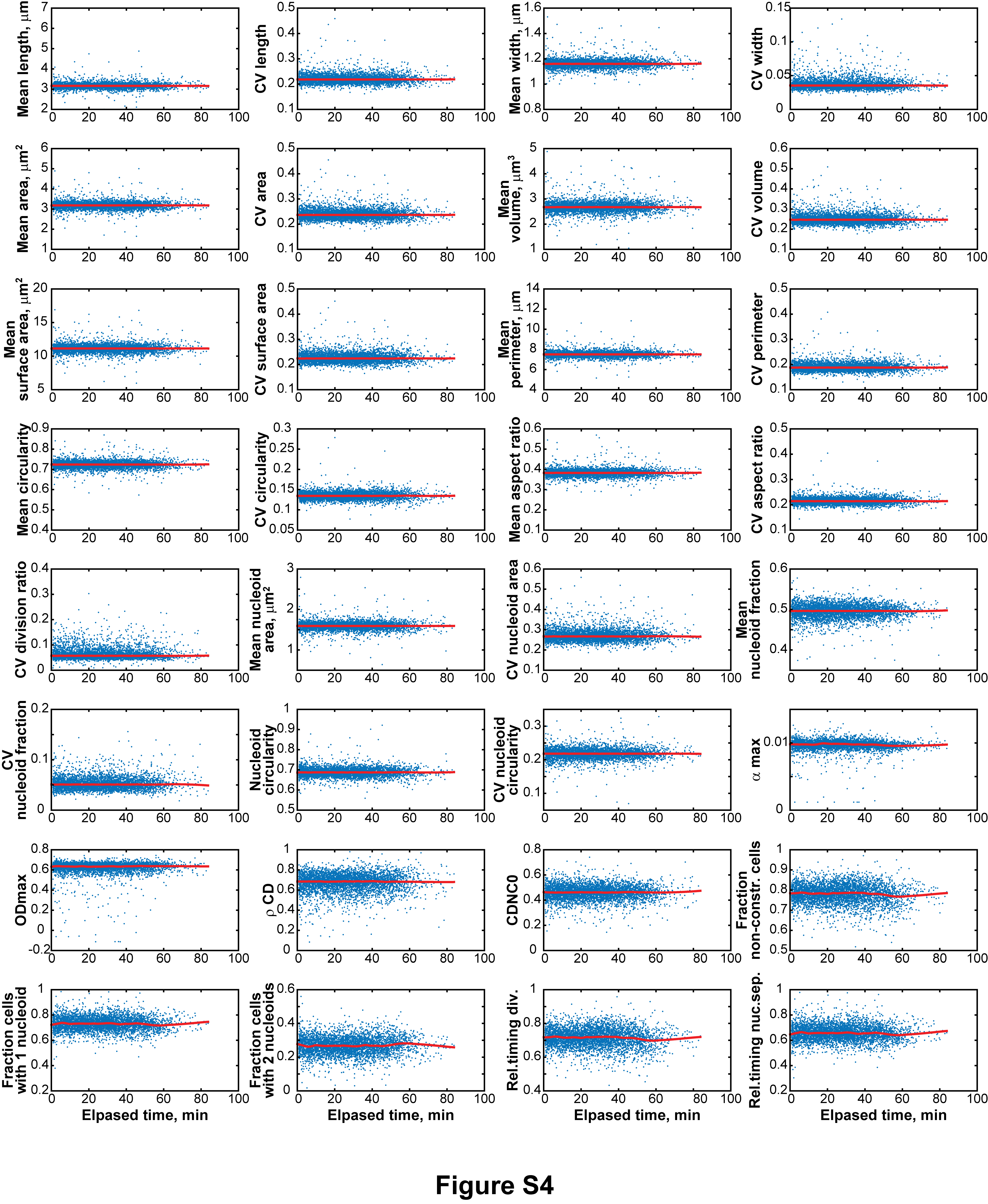


**Appendix Figure S4. Temporal detrending**. Scatter plot of the detrended data for all features. The red line in each graph represents the smoothing spline (calculated with a span of 20%). The flat profile for each of these average profiles illustrates the absence of temporal trend in the normalized data.


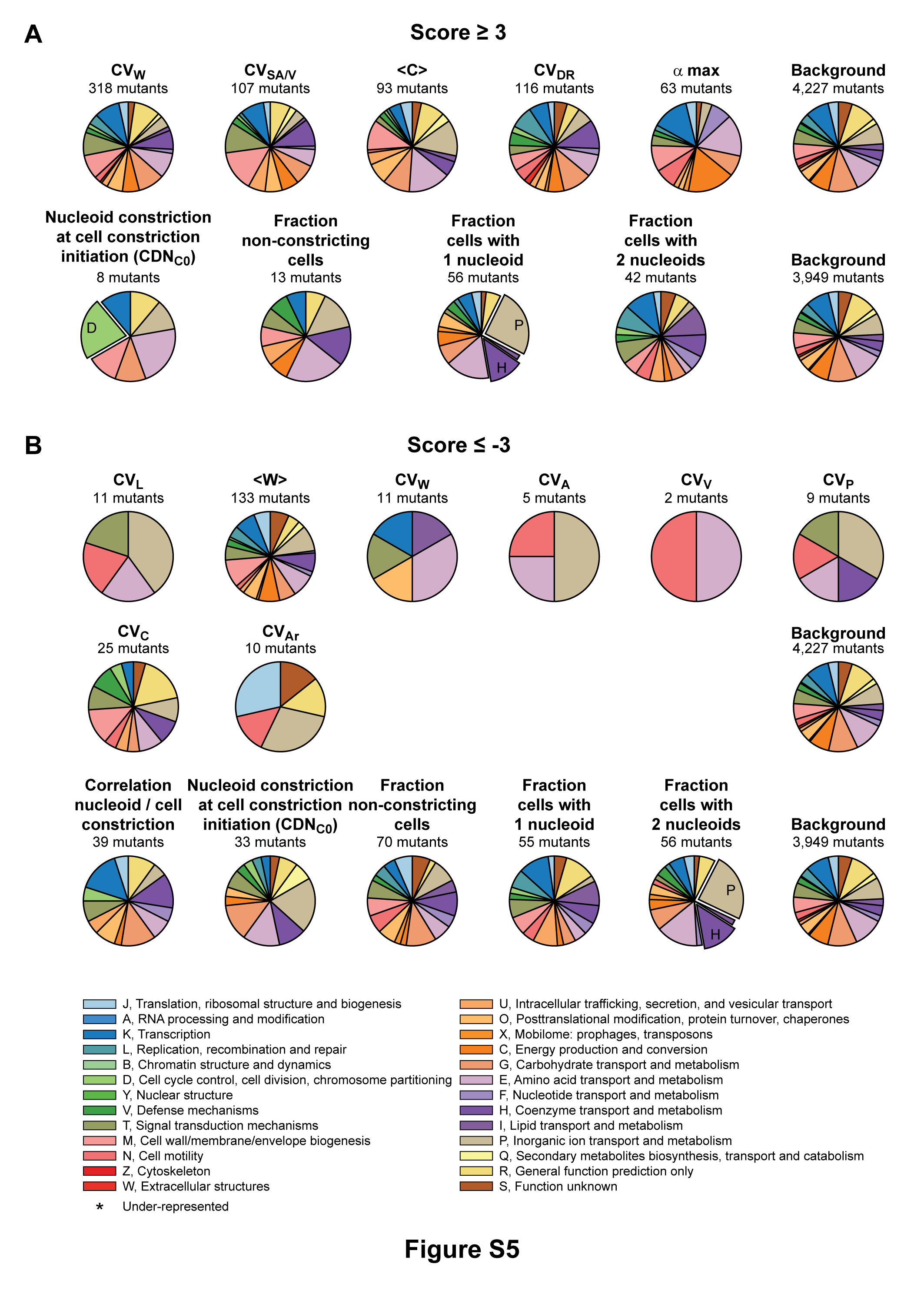


**Appendix Figure S5. Feature-based COG distribution analysis**. Pie charts representing, on a feature-by-feature basis, the relative distribution of COG categories among the gene deletion strains associated with a severe phenotype: **A.** *s* ≥ 3, **B.** *s* ≤ -3. All the features that were not included in Figure 3 are represented. The enriched COG categories are highlighted with an exploded pie sector. Enrichments with an associated (FDR corrected) q-value < 0.05 were considered significant.


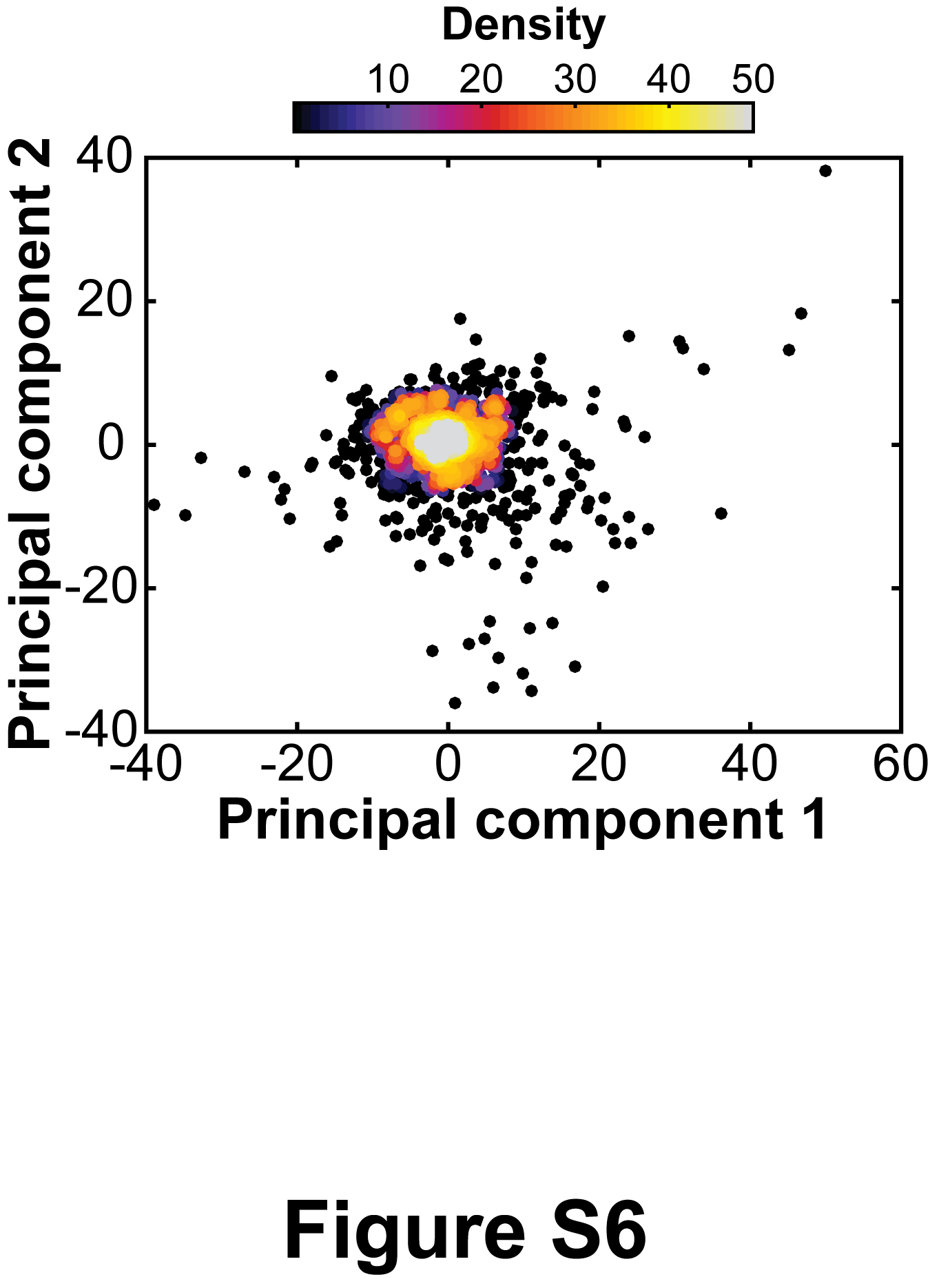


**Appendix Figure S6. Principal component analysis of the phenoprints associated with the morphological and growth mutants**. Scatter plot showing the coordinates of each strain in the first two principal components space. The density of points in any given area of the 2D space is illustrated by a color scale.
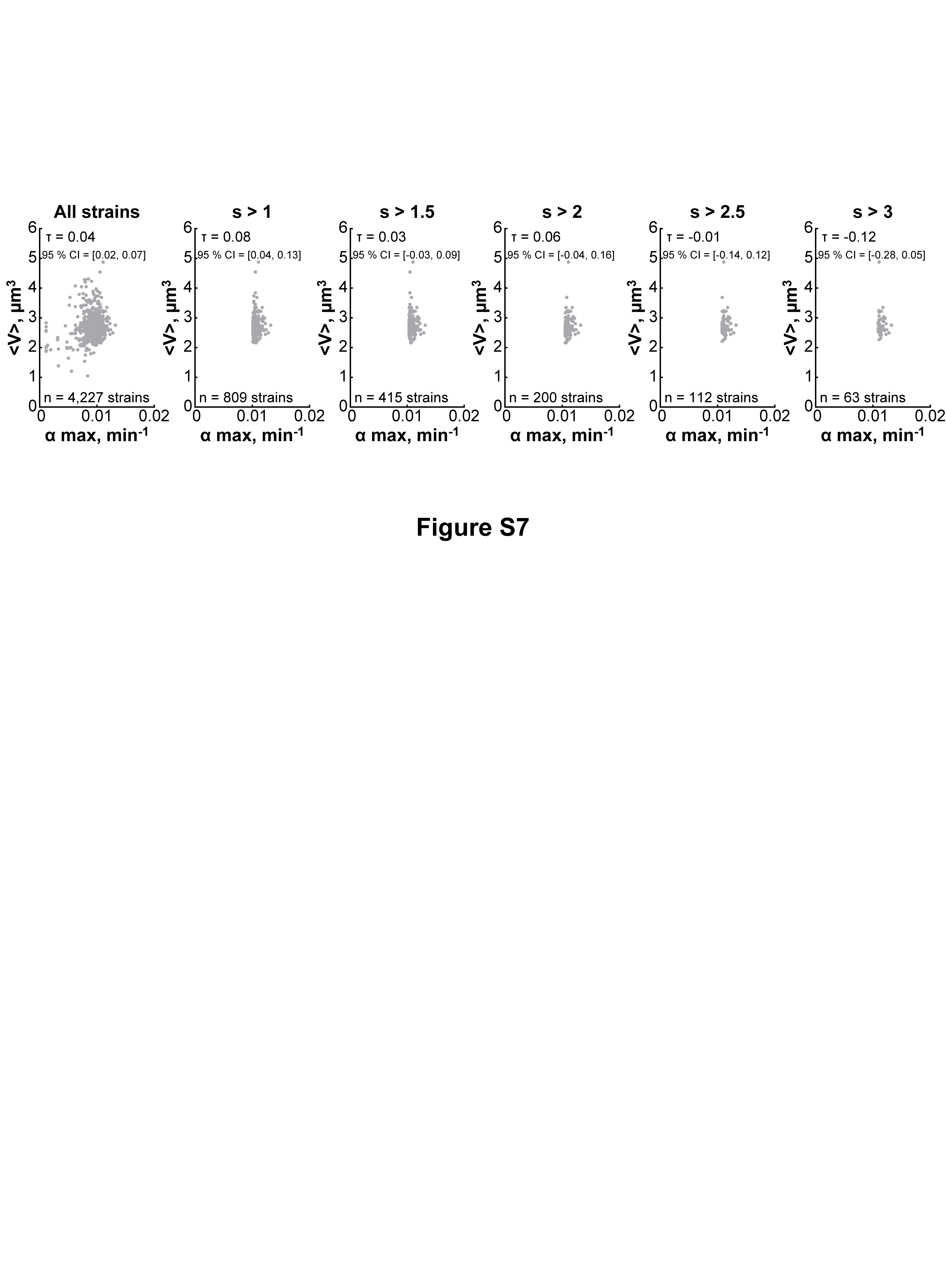


**Appendix Figure S7. Growth rate is not predictive of cell size even for fast-growing mutants**. Bootstrapped Kendall correlation values (5,000 samplings) between growth rate (α_max_) and mean cell volume (<V>) for all Keio strains (n = 4,227 strains) or for faster growing strains with α_max_ score > 1 (n = 809 strains), α_max_ score > 1.5 (n = 415 strains), α_max_ score > 2 (n = 200 strains), α_max_ score > 2.5 (n = 112 strains), α_max_ score > 3 (n = 63 strains).


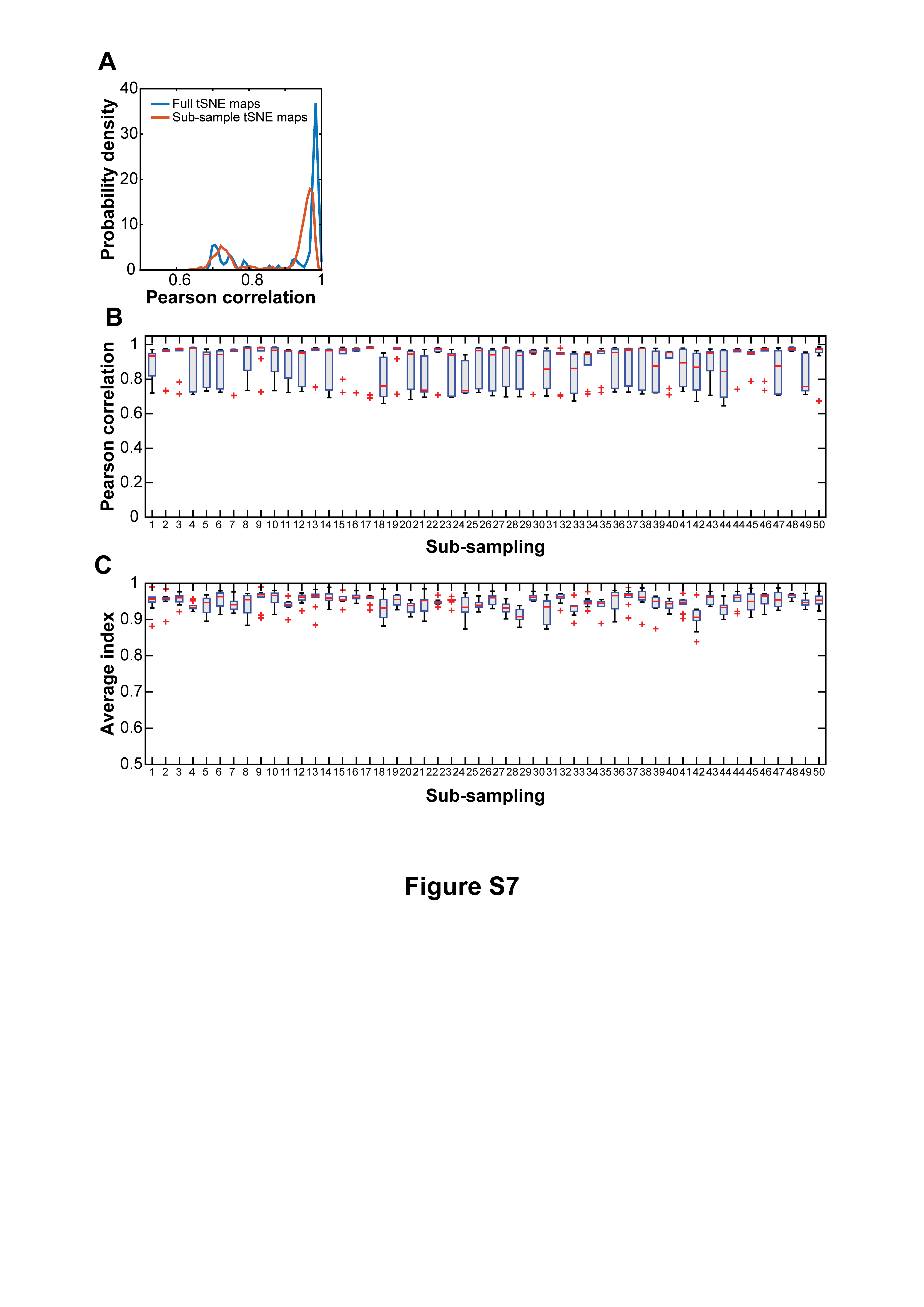


**Appendix Figure S8. Convergence of the dimensional reduction with tSNE**.

**A.** Probability density distribution of the Pearson correlation coefficients between pairwise distances of the points defined in the tSNE map represented in Fig 4A and those defined in 99 other tSNE maps for the 1,225 strains that exhibit a ∣s∣ > 3 for at least one morphological or growth feature (blue curve). We subsampled the original dataset in 50 disjoint sets of phenoprints, and generated 10 independent tSNE maps for each one. We then calculated the Pearson correlation coefficient between the tSNE coordinates of these points in each of these 500 tSNE maps with their corresponding coordinates in the tSNE map presented in Fig 4A. The orange curve represents the probability density distribution of these correlation values. The results show that all 500 tSNE maps are very similar despite their stochastic nature, indicating convergence.

**B.** Box plot showing the tight distributions of Pearson correlation coefficients between the pairwise distances between points for 10 independent tSNE maps calculated for each of the 50 sub-samples (500 tSNE maps) and their corresponding points in the tSNE map presented in Fig 4A. The blue boxes filled in gray represent the interquartile range, the red horizontal lines correspond to the median values, the whiskers cover the 2.7 standard deviation around the mean under a normality assumption, and the red crosses show the outliers beyond the limit of the whiskers.

**C.** Box plot showing the high degree of reproducibility of the dbscan clustering presented in Fig 4A. The dbscan algorithm was run on the subsample tSNE maps with the same parameters as for the full sample tSNE map (eps = 4.9, minPts = 3). The Jaccard index was calculated for each cluster using the clustering presented in Fig 4A as reference and all indexes were averaged over all clusters to score each of the 10 selected maps, providing 10 scores for each sub-sample. The blue boxes filled in gray represent the interquartile range, the red horizontal lines correspond to the median values, the whiskers cover the 2.7 standard deviation around the mean under a normality assumption, and the red crosses show the outliers beyond the limit of the whiskers.

# Appendix Tables

**Table S1. Features considered in this study and their associated symbols.** The aspect ratio was defined as the ratio of cell width over cell length at the single-cell level. The circularity, C, was defined as $C= \frac{4\pi A}{P^{2}}$, at the single-cell level, where P stands for perimeter and A for area. The relative timing of cell constriction and nucleoid separation were estimated as the proportions of cells without any significant constriction (constriction degree <0.15) or with a single nucleoid, respectively. For all cells with a significant constriction degree, we calculated the Pearson correlation coefficient between the constriction degrees of the cell and of its nucleoid (ρ CD). The nucleoid constriction degree at the initiation of cell constriction (CDN_C0_) was determined as the intercept of a line with a slope determined by the correlation coefficient that best fitted the single-cell data used to calculate ρ CD (see Appendix Fig S1E).

| Feature | Symbol |
| --- | --- |
| Morphological features |  |
| Mean cell length | <L> |
| Cell length variability | CV_L_ |
| Mean cell width | <W> |
| Cell width variability | CV_W_ |
| Mean cell area | <A> |
| Cell area variability | CV_A_ |
| Mean cell volume | <V> |
| Cell volume variability | CV_V_ |
| Mean cell surface area | <SA> |
| Cell surface area variability | CV_SA_ |
| Mean cell perimeter | <P> |
| Cell perimeter variability | CV_P_ |
| Mean circularity | <C> |
| Circularity variability | CV_C_ |
| Mean aspect ratio | <Ar> |
| Aspect ratio variability | CV_Ar_ |
| Mean surface area-to-volume ratio | <SA/V> |
| Surface area-to-volume ratio variability | CV_SA/V_ |
| Division ratio variability | CV_DR_ |
| Mean nucleoid area | <NA> |
| Nucleoid area variability | CV_NA_ |
| Growth features |  |
| Max growth rate | α_max_ |
| Optical density at growth saturation | OD_max_ |
| Cell cycle features |  |
| Correlation in nucleoid and cell constriction | ρ CD |
| Nucleoid constriction degree at the initiation of cell constriction | CDN_C0_ |
| Relative timing of cell constriction | Rel. timing div |
| Relative timing of nucleoid separation | Rel. timing nuc |
| Fraction of cells with 2 nucleoids | %2N |

**Table S2. Enriched GO terms in the morpho and cell cycle archipelagos.** GO term enrichments were assessed by neighborhoods (see Materials and methods). The table shows the enriched GO terms with a p-value adjusted for a false discovery rate below 0.05. A GO term can be enriched in multiple overlapping neighborhoods. The table lists, for each enriched GO term, the highest (least significant) adjusted p-value and the associated p-value that was calculated for all the overlapping neighborhoods where each GO term was enriched. The corresponding p-value is also reported. The color of the enrichment zone indicated in the “neighborhood” column refers to the color of the neighborhoods where the GO term is enriched in Fig 4E and Fig 5D. Finally, the type of ontology with which each GO term is associated is indicated in the “ontology” column.

| GO term | Highest p-value | Highest adjusted p-value | Neighborhood | Ontology |
| --- | --- | --- | --- | --- |
| Morpho archipelago |  |  |  |  |
| arginine biosynthetic process | 8.11E-11 | 6.49E-08 | dark blue | biological_process |
| cellular amino acid biosynthetic process | 3.55E-10 | 2.84E-07 | dark blue | biological_process |
| ATP biosynthetic process | 5.09E-08 | 4.65E-05 | grey | biological_process |
| ATP synthesis coupled proton transport | 2.19E-08 | 2.00E-05 | grey | biological_process |
| ion transport | 4.28E-06 | 3.92E-03 | grey | biological_process |
| plasma membrane ATP synthesis coupled proton transport | 7.32E-09 | 6.69E-06 | grey | biological_process |
| proton transport | 1.93E-06 | 1.76E-03 | grey | biological_process |
| proton-transporting ATP synthase activity, rotational mechanism | 2.19E-08 | 2.00E-05 | grey | molecular_function |
| proton-transporting ATPase activity, rotational mechanism | 2.19E-08 | 2.00E-05 | grey | molecular_function |
| biotin biosynthetic process | 3.89E-05 | 4.14E-02 | light blue | biological_process |
| FtsZ-dependent cytokinesis | 5.96E-06 | 4.24E-03 | orange | biological_process |
| cell division | 1.32E-05 | 2.82E-02 | red | biological_process |
| cysteine biosynthetic process | 3.70E-05 | 4.74E-02 | slate blue | biological_process |
| hydrogen sulfide biosynthetic process | 1.11E-06 | 1.02E-03 | slate blue | biological_process |
| sulfate assimilation | 2.50E-07 | 2.29E-04 | slate blue | biological_process |
| enterobacterial common antigen biosynthetic process | 5.03E-05 | 4.60E-02 | yellow | biological_process |
| Cell cycle archipelago |  |  |  |  |
| hydrogen sulfide biosynthetic process | 2.75E-06 | 2.09E-03 | blue | biological_process |
| sulfate assimilation | 1.16E-05 | 8.76E-03 | blue | biological_process |
| sulfite reductase complex (NADPH) | 4.83E-05 | 3.66E-02 | blue | cellular_component |
| sulfite reductase (NADPH) activity | 4.83E-05 | 3.66E-02 | blue | molecular_function |
| phosphate ion transmembrane transport | 2.75E-06 | 6.26E-03 | brown | biological_process |
| phosphate ion transport | 2.75E-06 | 6.26E-03 | brown | biological_process |
| ATPase-coupled phosphate ion transmembrane transporter activity | 2.75E-06 | 6.26E-03 | brown | molecular_function |
| arginine biosynthetic process | 1.36E-05 | 6.19E-03 | dark green | biological_process |
| cellular amino acid biosynthetic process | 6.11E-05 | 2.32E-02 | dark green | biological_process |
| biotin biosynthetic process | 6.47E-05 | 2.94E-02 | light blue | biological_process |
| ATP biosynthetic process | 4.71E-05 | 2.14E-02 | light green | biological_process |
| ATP synthesis coupled proton transport | 2.70E-05 | 1.23E-02 | light green | biological_process |
| ion transport | 6.47E-05 | 3.68E-02 | light green | biological_process |
| plasma membrane ATP synthesis coupled proton transport | 5.44E-07 | 1.24E-03 | light green | biological_process |
| proton transport | 6.47E-05 | 3.68E-02 | light green | biological_process |
| proton-transporting ATP synthase complex, coupling factor F(o) | 7.70E-06 | 1.75E-02 | light green | cellular_component |
| hydrogen ion transmembrane transporter activity | 7.70E-06 | 1.75E-02 | light green | molecular_function |
| proton-transporting ATP synthase activity, rotational mechanism | 2.70E-05 | 1.23E-02 | light green | molecular_function |
| proton-transporting ATPase activity, rotational mechanism | 2.70E-05 | 1.23E-02 | light green | molecular_function |
| enterobactin biosynthetic process | 7.70E-06 | 3.51E-03 | orange | biological_process |
| 2,3-dihydroxybenzoate-serine ligase activity | 7.70E-06 | 3.51E-03 | orange | molecular_function |

# Appendix Parameters

**Parameters S1. Parameters for cell identification in MicrobeTracker**. The parameters listed below were used to generate cell outlines in MicrobeTracker (Sliusarenko et al, 2011). These parameters can be copied and pasted into the MicrobeTracker parameter panel.

% This file contains MicrobeTracker %settings optimized for wildtype E. %coli

%cells at 0.064 um/pixel resolution %(using algorithm 4)

algorithm = 4

%Parallel Computation

runSerial = 0

maxWorkers = 12

%splitRegions

displayW = 0

wShedNum = 3800

% Pixel-based parameters

getmesh = 1

Nkeep = 320

areaMin =300

areaMax = 3000

scaleFactor = 1

thresFactorM = 0.974

thresFactorF = 0.974

splitregions = 1

edgedetection = 1

edgemode = 1

edgeSigmaL = 1.5

logthresh = 1

edgeSigmaV =0.5

valleythresh1 = 0.0002

valleythresh2 = 1

crossthresh = 0.15

repCoeff1 = 0

attrCoeff1 = 0

erodeNum = 0

opennum = 6

threshminlevel = 0.70

% Constraint parameters

fmeshstep = 1

meshstep = 1

cellwidth = 15

fsmooth = 100

imageforce = 8

wspringconst = 0

rigidityRange = 2.5

rigidity = 1

rigidityRangeB = 8

rigidityB = 5

attrCoeff = 0.2

repCoeff = 0.6

attrRegion = 4

horalign = 0.2

eqaldist = 2.5

% Image force parameters

fitqualitymax = 0.5

forceWeights = [0.25 0.65 0.25]

dmapThres = 2

dmapPower = 2

gradSmoothArea = 0.5

repArea = 0.9

attrPower = 4

neighRep = 5

% Mesh creation parameters

roiBorder = 22.5

noCellBorder = 2

maxmesh = 1000

maxCellNumber = 2000

maxRegNumber = 10000

meshStep = 1

meshTolerance = 0.01

meshWidth = 16

% Fitting parameters

erodeNum = 1

fitDisplay1 = 0

fitDisplay = 0

fitConvLevel = 0.26

fitMaxIter =500

fitMaxIter1 =500

moveall = 0.1

fitStep = 0.2

fitStepM = 0.6

% Joining and splitting

splitThreshold = 0.4

joindist = 5

joinangle = 0.2

joinWhenReuse = 1

split1 = 1

% Other

bgrErodeNum = 4

sgnResize = 1

aligndepth = 1

**Parameters S2. Parameters for nucleoid identification in Oufti**. The parameters listed below were used to generate nucleoid outlines using the objectDetection module in Oufti (Paintdakhi et al, 2016).

| Background subtraction method | 3 |
| --- | --- |
| Background subtraction threshold | 0..1 |
| Background filter size | 8 |
| Smoothing range (pixel) | 3 |
| Magnitude of LOG filter | 0.1 |
| Sigma o PSF | 1.62 |
| Fraction of object in cell | 0.4 |
| Minimum object area | 50 |

# References

Cooper S, Helmstetter CE (1968) Chromosome replication and the division cycle of *Escherichia coli* B/r. *J Mol Biol* **31:** 519-540

Maier MJ. (2014) DirichletReg: Dirichlet regression for compositional data in R. In Wien WU (ed.), *Research Report Series*. ePubWU Institutional Repository, WU Librayr, Vol. RVK SK 840, QH 234.

Paintdakhi A, Parry B, Campos M, Irnov I, Elf J, Surovtsev I, Jacobs-Wagner C (2016) Oufti: an integrated software package for high-accuracy, high-throughput quantitative microscopy analysis. *Mol Microbiol* **99:** 767-777

Sliusarenko O, Heinritz J, Emonet T, Jacobs-Wagner C (2011) High-throughput, subpixel precision analysis of bacterial morphogenesis and intracellular spatio-temporal dynamics. *Mol Microbiol* **80:** 612-627

Wang X, Lesterlin C, Reyes-Lamothe R, Ball G, Sherratt DJ (2011) Replication and segregation of an *Escherichia coli* chromosome with two replication origins. *Proc Natl Acad Sci USA* **108:** E243-250
