# Supplementary material for: Genomewide phenotypic analysis of growth, cell morphogenesis, and cell cycle events in Escherichia coli
Source: Mol Syst Biol. 2018 Jun 25;14(6):e7573. doi: 10.15252/msb.20177573 (PMC6018989; doi:10.15252/msb.20177573)
Supplement: Supplementary file 2 — Expanded View Figures PDF [file MSB-14-e7573-s002.pdf]

Expanded View Figures

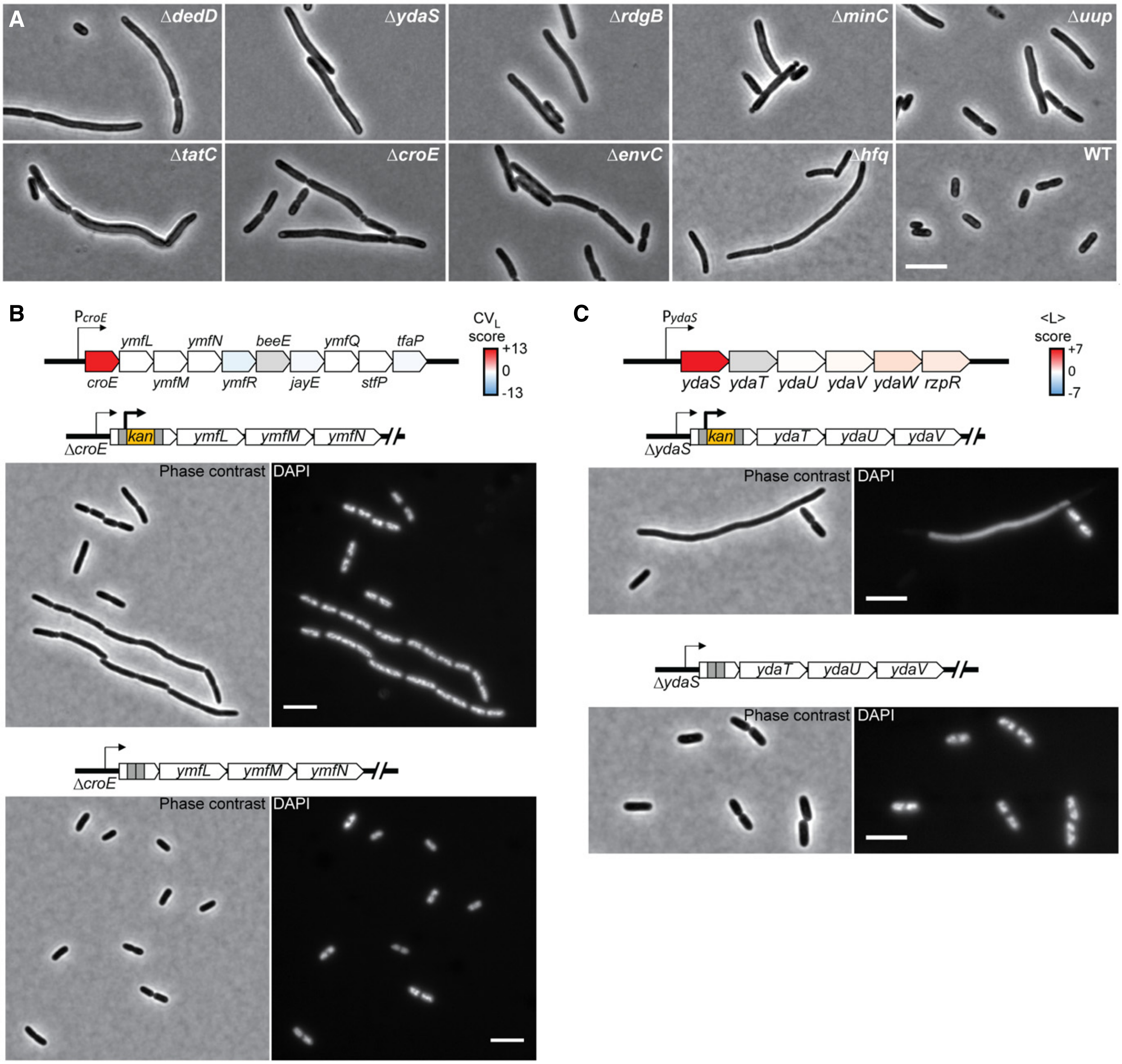

Figure EV1. Filamentous mutants.

A Representative phase-contrast images of the mutants forming the island 22, together with the parental strain BW25113 (WT) for comparison. Scale bar corresponds to 5 μm.

B Effect of the kanamycin-resistance cassette on the phenotype of the Δ*croE* strain. The schematic at the top shows the color-mapped score of CV of cell length for the deletion of each gene of the *croE* operon. Below are phase-contrast and fluorescent images of DAPI-stained cells of the Δ*croE* strain carrying the kanamycin-resistance cassette (top) or after the removal of the cassette (bottom). The *ymfN* locus has been re-annotated as two separate genes (*oweE* and *aaaE*), and the Keio deletion strain of *ymfN* carries the deletion of these two contiguous genes. Scale bars correspond to 5 μm.

C Effect of the kanamycin-resistance cassette on the phenotype of the Δ*ydaS* strain. The schematic at the top shows the color-mapped score of the mean cell length for each gene of the *ydaS* operon. Below are phase-contrast and fluorescent images of DAPI-stained cells of the Δ*ydaS* strain carrying the kanamycin-resistance cassette (top) or after the removal of the cassette (bottom). Scale bars correspond to 5 μm.

© 2018 The Authors

Figure EV2. Specific pathways associated with impaired cell morphology.

A Schematic of the ECA biosynthetic pathway in which each gene name has been colored by the severity of the mean circularity (<C>) phenotype.

B Scatter plot of cell width versus cell length for three independent liquid cultures of the Δ*rapZ* strain (*n* = 564, 268 and 343 cells) in well-agitated test tubes. The dotted lines represent isocontours of a 2D histogram of cell length and cell width for the parental strain (WT, *n* = 1,045 cells). The cell width distributions of the WT and Δ*rapZ* strains are represented on the right of the scatter plot (all three replicates for the Δ*rapZ* strain were pooled together).

C Schematics of the ATP synthase, the high-affinity ABC phosphate transporter, and its related proteins. Proteins and subunits have been colored according to the severity of the mean cell width (<W>) phenotype in the corresponding gene deletion strains.

D Bar graph showing the mean cell length (<L>), width (<W>), and area (<A>) scores for the Δ*fadR* and Δ*fabF* deletion strains. The scores for saturated and unsaturated phospholipid metabolites detected in these strains by Fuhrer *et al* (2017) are represented as a heatmap.

© 2018 The Authors

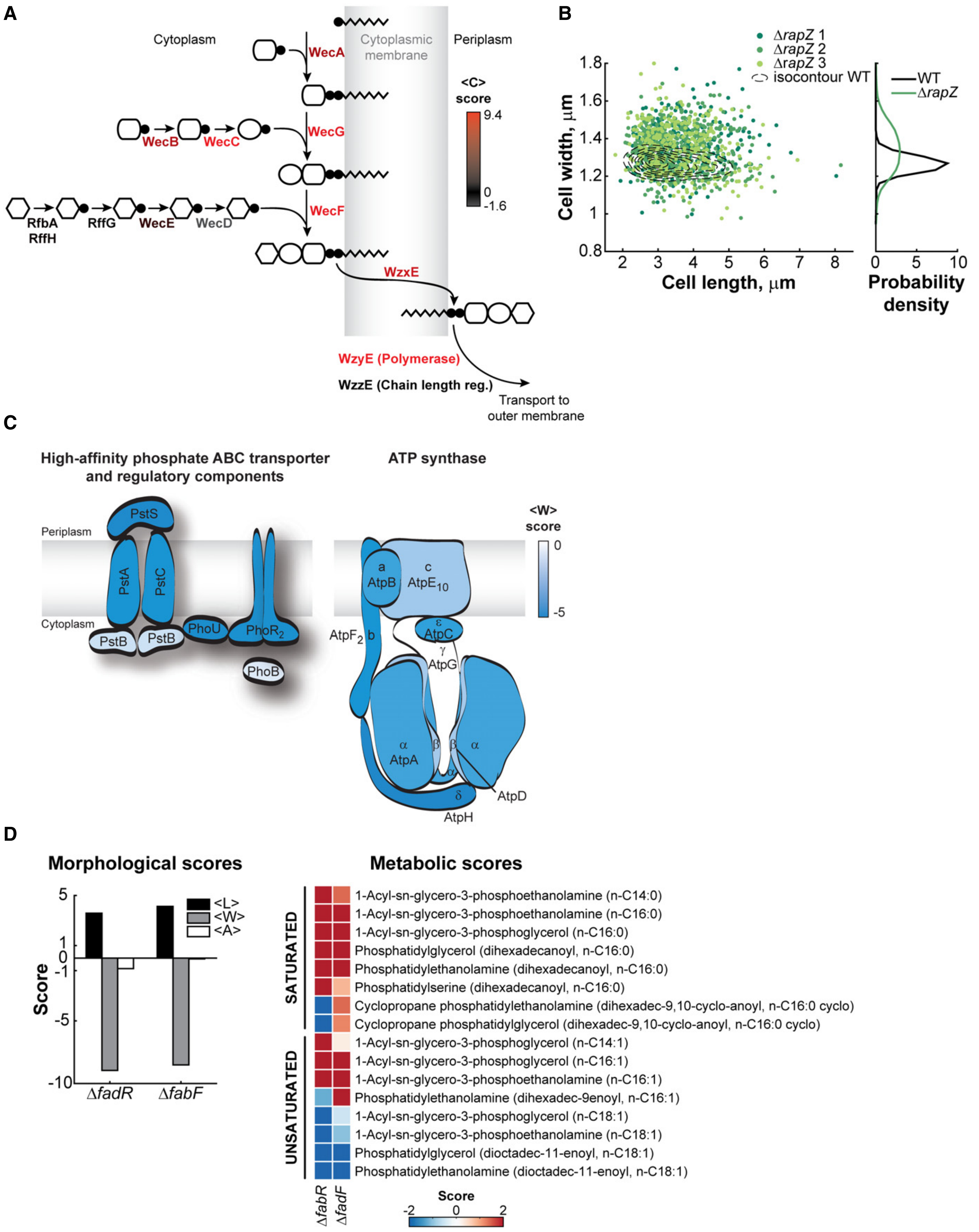

Figure EV2.

© 2018 The Authors

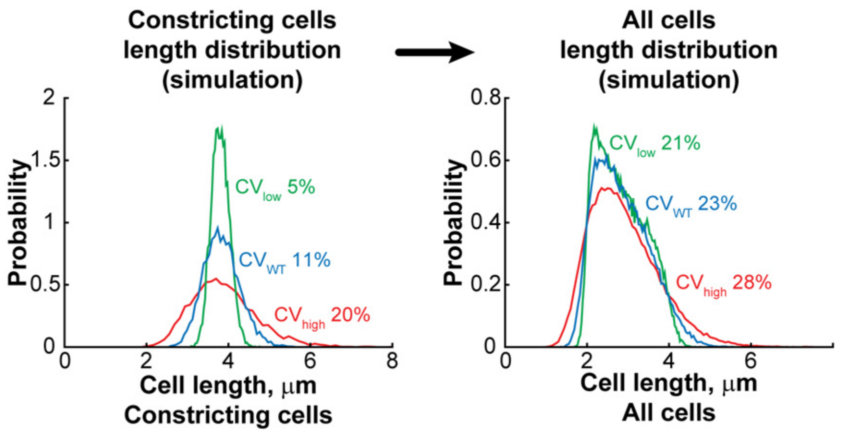

Figure EV3. Simulation showing that the cell length variability of the entire population can mask abnormal cell length variability at a specific cell cycle period.

Cell length distributions were simulated over different ranges of cell ages (see Materials and Methods). The cell length distribution of constricting cells was determined by summing the cell length distributions of all cells of age > 0.8, assuming different CV of the cell length distribution (0.05, 0.11, and 0.2) at a specific age. The cell length distribution of the whole population was determined by summing the distributions at all ages, from birth to division.

© 2018 The Authors

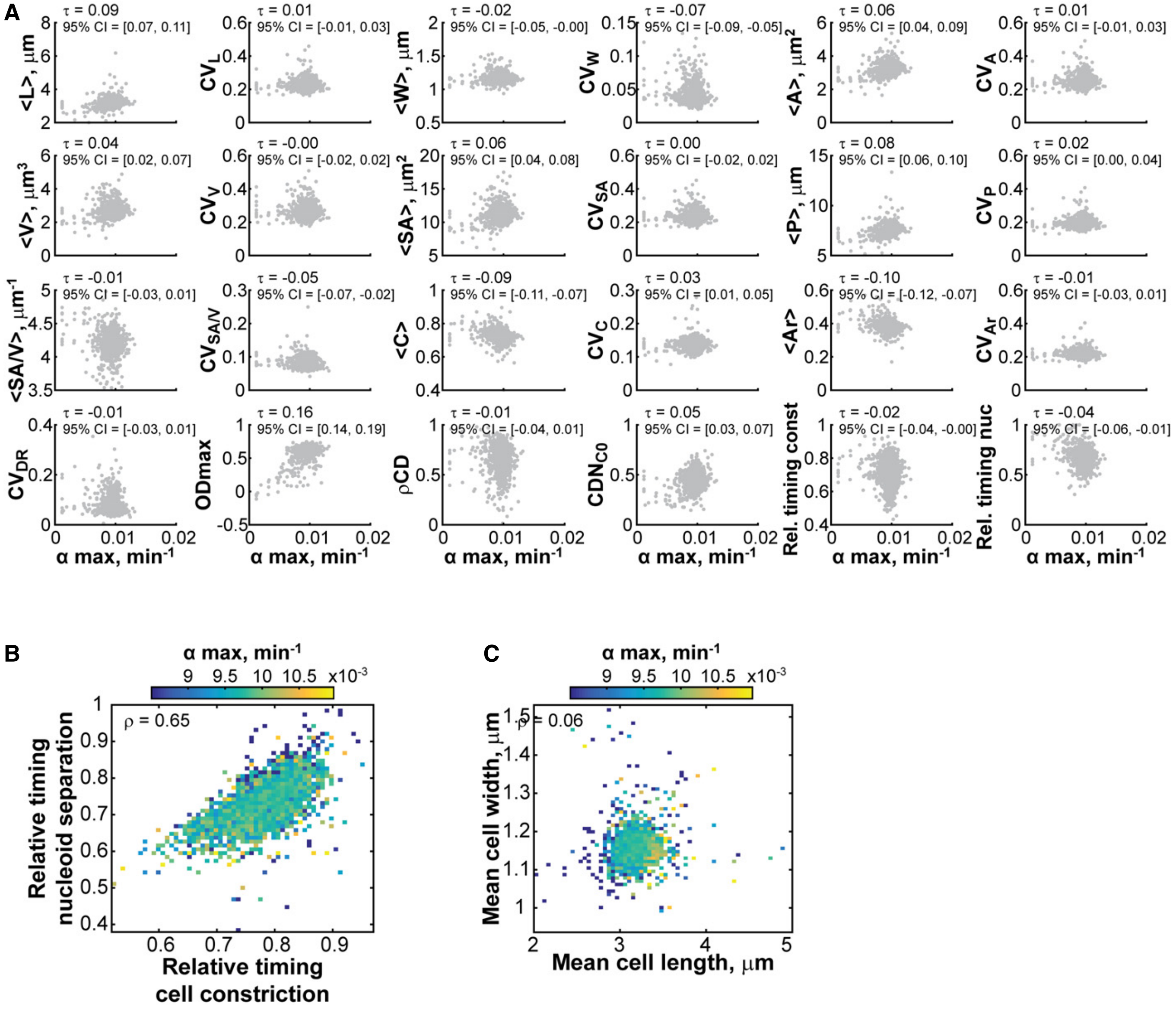

Figure EV4. Growth rate correlates poorly with morphological and cell cycle features.

A Scatter plots of morphological and cell cycle features versus α_{max}. Each gray dot represents one Keio strain. The Kendall correlation coefficient τ and the associated 95% confidence interval are reported for each pair of features. The confidence intervals were calculated by bootstrapping the correlation 5,000 times and taking the 2.5 and 97.5 percentiles of the resulting distribution. The Kendall rank correlation τ was selected over Pearson correlation because of the heavily asymmetric left tail in the distribution of α_{max}.

B Heatmap showing the mean growth rate value for data binned by the relative timings of cell constriction and nucleoid separation.

C Same as in (B), except for data binned by mean cell length and mean cell width.

 © 2018 The Authors

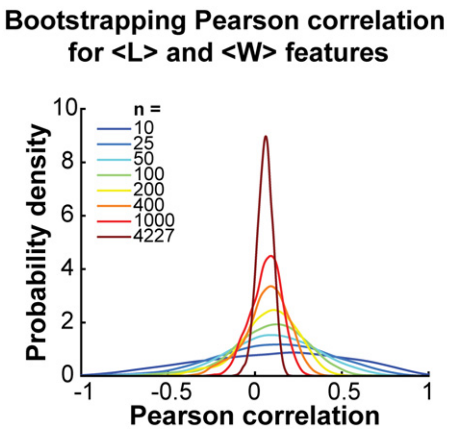

Figure EV5. The sample size affects the confidence in the estimation of the correlation coefficient.

The scores for the <L> and <W> features were sampled 5,000 times independently with replacement with different sample sizes ($n = 10$, 25, 50, 100, 200, 400, 1,000, or 4,227), and the Pearson correlation coefficient was calculated at each sampling. The distribution of the 5,000 correlation values obtained for each sample size is represented with a different color. The probability density distributions were estimated using a kernel density estimation method (Botev *et al*, 2010). The plot shows that a small sampling size, such as $n = 10$, results in a wide distribution of possible Pearson correlations. Increasing the sample size narrows down the distribution, increasing the confidence in the obtained correlation value.

© 2018 The Authors
